# A Rare Case of Myxochondroid Metaplasia of the Plantar Foot

**DOI:** 10.7759/cureus.90118

**Published:** 2025-08-14

**Authors:** Michael C Elias, Matthew C Elias, Melissa P Piliang, Christine C Tam

**Affiliations:** 1 Dermatology, Certified Dermatologists, North Royalton, USA; 2 Dermatology, Cleveland Clinic Foundation, Cleveland, USA; 3 Dermatology, Southwest General Health Center, Middleburg Heights, USA

**Keywords:** cartilaginous tumor, fibrocatilaginous pseudotumor, mechanical stress, myxochondroid metaplasia, plantar foot, pseudoneoplastic lesion, soft-tissue lesion

## Abstract

We present a case of a 52-year-old male with a slowly enlarging, asymptomatic nodule on his heel diagnosed as myxochondroid metaplasia of the plantar foot. The incidence is rare, as this is only the second case reported since it was first discovered. It is a benign tumor believed to represent a reaction to chronic mechanical stress, presenting as a variably painful, skin-colored nodule on the plantar foot. Histologically, a subepidermal nodule comprised of spindled fibroblasts and cartilaginous elements is seen. It is histopathologically reminiscent of the nuchal fibrocartilaginous pseudotumor as well as the acquired equine digital cushion, two other reactive fibrocartilaginous growths in the neck and a horse’s hoof, respectively. Excision is curative.

## Introduction

Nuchal fibrocartilaginous pseudotumor is an uncommon soft-tissue lesion occurring within the nuchal ligament at the posterior midline of the neck. It is thought to arise through fibrocartilaginous metaplasia in previously traumatized nuchal ligament tissue [1]. Recently, a unique entity reminiscent of the nuchal fibrocartilaginous pseudotumor has been described on the plantar foot, occurring as a reactive process due to chronic mechanical stress. It presents as a variably painful nodule on the plantar foot that is comprised of spindled fibroblasts and cartilaginous elements histopathologically. Authors termed this condition myxochondroid metaplasia of the plantar foot [2]. We report the second case of this rare pseudoneoplastic lesion since it was first described.

## Case presentation

**Figure 1:**
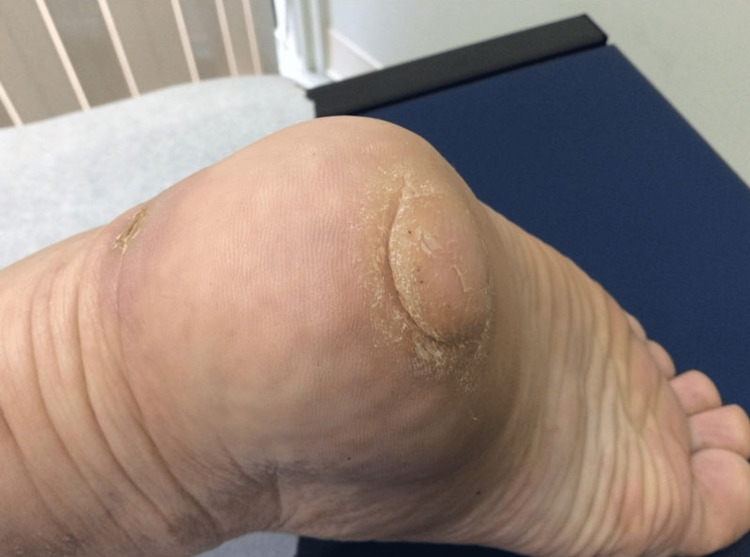
Clinical photo Firm, skin-colored nodule involving the right plantar heel.

A 52-year-old male presented with a 15-year history of a slowly enlarging, asymptomatic lesion on the right plantar foot. He had a sedentary job and hobbies, and he denied any trauma to the region. The lesion appeared as a firm, skin-colored, dome-shaped, 3.0-cm nodule involving the right plantar heel (Figure 1).

**Figure 2:**
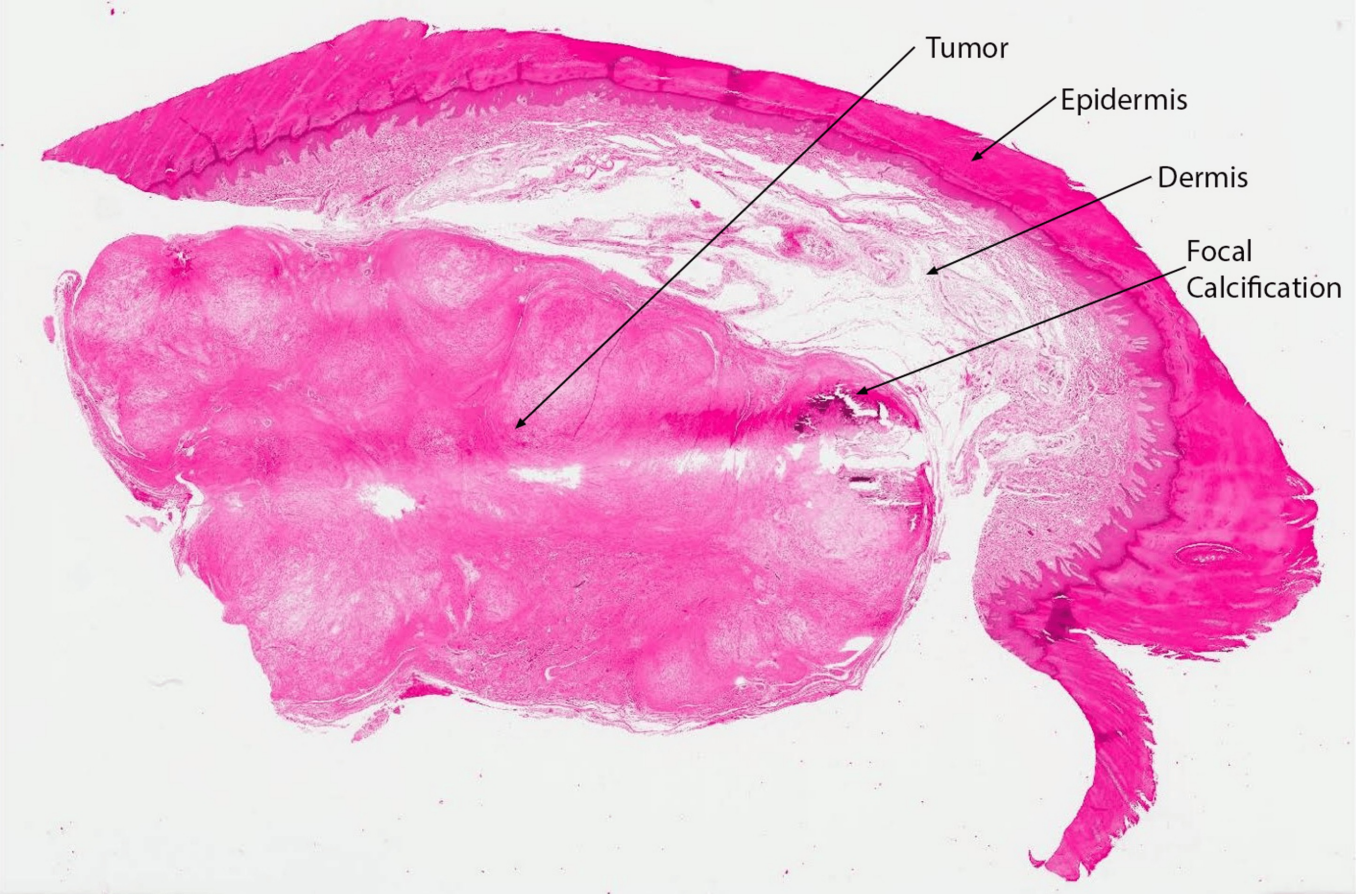
Biopsy photo, hematoxylin-eosin, original magnification x20 Circumscribed lobulated tumor, with focal calcification, within the dermis

**Figure 3:**
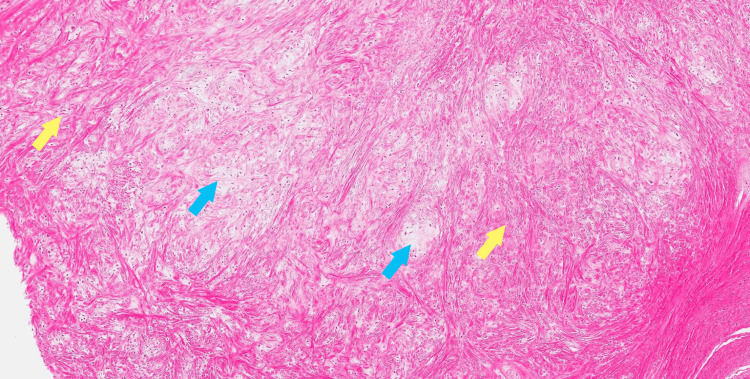
Biopsy photo, hematoxylin-eosin, original magnification x100 Hypocellular spindled fibroblasts (yellow arrows) with cartilaginous elements in myxoid stroma (blue arrows)

The lesion was excised. Grossly, the specimen appeared as an irregularly shaped segment of skin, with an attached subcutaneous nodule measuring 3.6 x 2.7 x 2.5 cm. Sectioning revealed a white, waxy, and chalky cut surface. Histologic sections demonstrated a circumscribed lobulated tumor within the dermis comprised of hypocellular, bland spindled fibroblasts and cartilaginous elements with focal calcification (Figures 2, 3).

The patient was diagnosed with myxochondroid metaplasia of the plantar foot (fibrocartilaginous pseudotumor). At his follow-up 14 months later, the site was well-healed with no evidence of recurrence. He remains recurrence-free at 9 years of follow-up.

## Discussion

Myxochondroid metaplasia of the plantar foot was first described in a series of nine patients by Shon and Folpe in 2013 [2]. Myxochondroid metaplasia is a rare, distinctive, chondroid soft-tissue lesion arising on the plantar aspect of the foot that is thought to represent a reactive process to chronic mechanical stress. Patients aged 8 to 78 years old of both sexes have been affected. The reported lesions all occurred in the soft tissue of the plantar foot and were variably painful. Histologically, lesions were partially circumscribed, sub-epidermal nodules of bland fibroblastic cells in a fibromyxoid background with vaguely to distinctly cartilaginous areas. By immunohistochemistry, variable expression of S100 and ERG proteins was noted, confirming cartilage differentiation. No recurrences were observed after excision [2,3].

The morphological features of myxochondroid metaplasia of the plantar foot are similar to those of the nuchal fibrocartilaginous pseudotumor first described by O’Connell et al in 1996. Nuchal fibrocartilaginous pseudotumor presents as a soft-tissue mass in the posterior base of the neck. Histologically, it consists of a fibrocartilaginous nodule located in the nuchal ligament. Often occurring after trauma from a motor vehicle accident, it is thought to develop as a reaction to soft-tissue injury in a manner similar to fibrocartilaginous metaplasia seen in degenerated tendoligamentous structures [1]. The histological features of myxochondroid metaplasia of the plantar foot are also reminiscent of the acquired digital equine cushion. This structure helps to absorb physical shock and is present only in exercised horses as a reaction to constant mechanical stress. It consists of fibrocartilage, myxoid tissue, connective tissue, and fat [2].

Myxochondroid metaplasia of the plantar foot must be distinguished from other soft-tissue tumors containing a cartilaginous component histopathologically. Chondromas contain well-defined lobules of mature chondrocytes in a cartilaginous matrix [3]; fibro-osseous pseudotumors of the digits contain a myofibroblastic component and bone production in addition to cartilaginous tissue; tumoral calcinosis consists of aggregates of calcification surrounded by histiocytes; calcifying aponeurotic fibroma contains an infiltrative, cellular fibroblastic component in addition to islands of variably calcified, chondroid matrix; and synovial chondromatosis arises from synovium tissue [2].

## Conclusions

We have reported the second case of myxochondroid metaplasia of the plantar foot since it was first described in a series of nine patients. This is a rare pseudoneoplastic lesion composed of fibroblastic and cartilaginous elements occurring on the plantar foot due to chronic mechanical stress. It resembles the nuchal fibrocartilaginous pseudotumor as well as the acquired equine digital cushion. This condition should be considered in the differential diagnosis of a plantar nodule.
